# Production of Mass-Separated Erbium-169 Towards the First Preclinical *in vitro* Investigations

**DOI:** 10.3389/fmed.2021.643175

**Published:** 2021-04-22

**Authors:** Zeynep Talip, Francesca Borgna, Cristina Müller, Jiri Ulrich, Charlotte Duchemin, Joao P. Ramos, Thierry Stora, Ulli Köster, Youcef Nedjadi, Vadim Gadelshin, Valentin N. Fedosseev, Frederic Juget, Claude Bailat, Adelheid Fankhauser, Shane G. Wilkins, Laura Lambert, Bruce Marsh, Dmitry Fedorov, Eric Chevallay, Pascal Fernier, Roger Schibli, Nicholas P. van der Meulen

**Affiliations:** ^1^Center for Radiopharmaceutical Sciences ETH-PSI-USZ, Paul Scherrer Institute, Villigen, Switzerland; ^2^Department of Chemistry and Applied Biosciences, ETH Zurich, Zurich, Switzerland; ^3^Laboratory of Radiochemistry, Paul Scherrer Institute, Villigen, Switzerland; ^4^European Organization for Nuclear Research (CERN), Geneva, Switzerland; ^5^Institute for Nuclear and Radiation Physics, Catholic University of Leuven, Leuven, Belgium; ^6^Institut Laue-Langevin, Grenoble, France; ^7^Institute of Radiation Physics, University Hospital and University of Lausanne, Lausanne, Switzerland; ^8^Institute of Physics, Johannes Gutenberg University, Mainz, Germany; ^9^Institute of Physics and Technology, Ural Federal University, Yekaterinburg, Russia; ^10^Analytic Radioactive Materials, Paul Scherrer Institute, Villigen, Switzerland; ^11^Petersburg Nuclear Physics Institute, National Research Center Kurchatov Institute, Gatchina, Russia

**Keywords:** Er-169, electromagnetic isotope separation, lanthanide-separation, activity standardization, *in vitro* studies, laser resonance ionization

## Abstract

The β^−^-particle-emitting erbium-169 is a potential radionuclide toward therapy of metastasized cancer diseases. It can be produced in nuclear research reactors, irradiating isotopically-enriched ^168^Er_2_O_3_. This path, however, is not suitable for receptor-targeted radionuclide therapy, where high specific molar activities are required. In this study, an electromagnetic isotope separation technique was applied after neutron irradiation to boost the specific activity by separating ^169^Er from ^168^Er targets. The separation efficiency increased up to 0.5% using resonant laser ionization. A subsequent chemical purification process was developed as well as activity standardization of the radionuclidically pure ^169^Er. The quality of the ^169^Er product permitted radiolabeling and pre-clinical studies. A preliminary *in vitro* experiment was accomplished, using a ^169^Er-PSMA-617, to show the potential of ^169^Er to reduce tumor cell viability.

## Introduction

Radiolanthanides are of particular interest in the field of nuclear medicine, offering attractive decay properties for both diagnosis and therapy (1–6). One of the most intriguing features of the radiolanthanides, other than having promising physical decay properties, is that they have similar chemical characteristics, and analogous coordination chemistry, which allows one to perform comparative pre-clinical studies. A significant disadvantage of lanthanides, however, is that they are difficult to chemically separate and isolate.

Currently, the β^−^-emitting ^177^Lu is used on a routine basis in clinics for targeted radionuclide therapy. The Center of Radiopharmaceutical Sciences (CRS) at Paul Scherrer Institute (PSI) has recently introduced ^161^Tb as a potentially better alternative to ^177^Lu due to its co-emission of conversion and Auger electrons (Table 1). There is good reason to assume that conversion and Auger electrons emitted by ^161^Tb, in addition to β^−^-particles, have an additive therapeutic effect (7). However, further studies are needed to investigate the contribution of conversion and Auger electrons in more detail. In this regard, the use of radionuclides with either β^−^- or Auger-electron emission would be ideal to investigate. The matched pair of ^169^Er (pure β^−^-particle emitter) with the pure Auger-electron emitter ^165^Er can, thus, represent an ideal model system to evaluate the additive therapeutic effect of Auger electrons on targeted β^−^ therapy.

**Table 1:** Comparison of decay properties and Auger electron/Conversion electron energies of ^161^Tb, ^169^Er, and ^165^Er, respectively.

**Radionuclide**	**t_**1/2**_**	**$\beta_{\text{av}}^{-}$ (keV)**	**Electrons**		
				**Energy (keV)**	**Intensity (%)**
^161^Tb	6.96 d	154	CE K	3.4	17.5
			Auger L	5.2	87.9
			CE L	16.6	41.0
			CE K	20.8	5.7
			CE M	23.6	9.2
^169^Er	9.39 d	100	CE M	6.1	36.0
			CE N	7.9	8.2
^165^Er	10.36 h	_	Auger L	5.3	65.6
			Auger K	38.4	4.8

*Only the high intensities are shown (data are taken from ^1^)*.

^169^Er (*t*_1/2_ = 9.39 d, E$\beta_{\text{av}}^{-}$ = 100 keV^1^) and ^165^Er (*t*_1/2_ = 10.36 h) have promising decay properties toward radionuclide therapy of metastasized cancer diseases (Table 1). Dosimetry calculations showed that ^169^Er displayed higher tumor-to-normal-tissue absorbed dose ratio (TND) values than the currently clinically-used radiolanthanide ^177^Lu (8) for tumor sizes from 10^2^ to 10^−9^ g (3), which is ascribed to its soft β^−^-radiation and the negligible photon dose (only 0.017 eV per decay^1^). Its low-energy β^−^-particles have an average soft tissue range of 0.3 mm (9). Moreover, the relatively long half-life of ^169^Er is considered as an additional advantage to avoid activity loss during transport and storage. On the other hand, ^165^Er is an attractive radiolanthanide for pure Auger-electron therapy. It decays by electron capture, followed by the emission of Auger electrons without accompanying γ-radiation, but emission of X-rays with an average energy of 48.8 keV. Systematic pre-clinical studies, by combining ^169^Er and ^165^Er with varying activity ratios, would help in understanding the additive therapeutic effect of Auger electrons, which requires the availability of these radionuclides in a suitable quality for pre-clinical studies.

Erbium-169 is currently produced by neutron irradiation of highly-enriched (98.2%) ^168^Er_2_O_3_ targets in nuclear reactors. In this way, it can only be produced with low specific activity in the carrier-added form due to the relatively low neutron capture cross-section of ^168^Er (σ: 2.3 barn) (10). To date, ^169^Er has only been used as a colloid in citrate form for the treatment of chronic rheumatoid arthritis, inflamed synovium by means of irradiation and to improve joint function (9, 11–18). For this purpose, low-specific activities are acceptable. However, for tumor-targeted radionuclide therapy, ^169^Er is required as a high-specific activity product.

A combination of mass and chemical separation, to obtain high-purity radionuclides for nuclear medicine applications, represents an innovative approach to achieve this goal (19–21). As proof-of-principle experiments, the ISOLDE (Isotope Separation On-Line) facility at CERN was used for the mass separation of medically-researched terbium radionuclides (22–25). Neutron-deficient diagnostic and therapeutic terbium radionuclides [^152^Tb (19, 20, 26–29), ^155^Tb (27, 30), and ^149^Tb (21, 27, 31, 32)], were separated at ISOLDE and, after chemical separation at PSI, utilized for pre-clinical *in vitro* and *in vivo* studies. Similar mass separation techniques have recently been used at the CERN-MEDICIS facility with off-line mass separation capabilities (33–40). An ^169^Er mass separation proof of concept (up to 17 MBq), obtained from reactor-irradiated enriched ^168^Er targets, was previously performed using an electromagnetic isotope separation (EMIS) method (37).

In the present work, an offline mass separation technique was used in combination with a resonance laser ion source (34, 39, 41, 42), which ensured the increase of efficiency and selectivity for ^169^Er. The activities produced were sufficient to perform one preliminary *in vitro* assay. Previously, ionization efficiency values in the order of 20–50% (on mass separators equipped with laser ion sources) were reported for holmium, dysprosium and lutetium (38, 43, 44). Similar results were also obtained at the CERN-MEDICIS facility for ^167^Tm (45), indicating room for separation efficiency improvements for ^169^Er. In addition to higher separation efficiencies, longer irradiation times and minimum activity loss during radiochemical separation and transport will allow one to produce ^169^Er in much higher activities for comprehensive pre-clinical studies and, to evaluate the potential of this novel radionuclide for future clinical applications.

**Figure 1:**
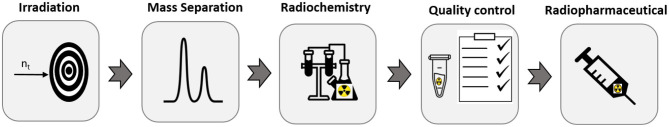
Stepwise production of ^169^Er for receptor-targeted radionuclide therapy (chemical set up image was taken from https://thenounproject.com/ under the license number: ch_0GVAv9Ol-QUEvnWfJrWcOvos9).

This systematic study consisted of several steps, namely, irradiation, mass separation, radiochemistry, quality control, and radiolabeling, to obtain a standard procedure for the production of ^169^Er in view of its use for receptor-targeted radionuclide therapy (Figure 1). After the mass separation process, the subsequent chemical separation method was developed to obtain ^169^Er in a solution of sufficient quality to enable radiolabeling of tumor-targeting agents. The activity standardization of ^169^Er was completed using the triple-to-double-coincidence ratio (TDCR) technique to perform precise activity measurements (46). A preliminary *in vitro* experiment was carried out to assess tumor cell viability upon exposure to ^169^Er-labeled PSMA-617 using cancer cells expressing the prostate-specific membrane antigen (PSMA).

## Materials and Methods

### Production of Carrier-Added ^169^Er

Enriched ^168^Er_2_O_3_ (98.0%, ISOFLEX, USA and 98.2% Trace Sciences Int., Canada) was used as target material for the production of ^169^Er *via* the ^168^Er(*n*, γ)^169^Er nuclear reaction. The ^168^Er_2_O_3_ samples (7.9–14.2 mg) were sealed in quartz ampoules and irradiated in the V4 irradiation position of the high-flux reactor at Institute Laue–Langevin (ILL), Grenoble, France (thermal neutron flux ≈ 1.1 10^15^ n.cm^2^.s^−1^, irradiation time: 7 days, ^169^Er theoretical yield: 25-48 GBq). After irradiations, the ampoules were transported to the CERN-MEDICIS facility for the offline mass separation of *A* = 169.

Carrier-added ^169^Er, supplied as a colloidal suspension of ^169^Er-citrate from Curium (Swiss distributor: b.e. imaging GmbH), was used as a representative sample for post-irradiation and after chemical separation for activity standardization. The list of all the characterized samples, using different techniques, is given in Table 2.

**Table 2:** The list of the used and characterized samples.

**Samples**	**Characterization**
Enriched ^168^Er_2_O_3_ (Isoflex)	ICP-MS
Enriched ^168^Er_2_O_3_ (Trace Sciences)	ICP-MS
Carrier-added ^169^Er (b.e. Imaging)	Activity standardization (TDCR)
Seven samples after mass separation	γ-ray spectrometry
Seven samples after mass and chemical separation	Quality control analyses (section Quality Control)

### Mass Separation

MEDICIS' Laser Ion Source for Separator Assembly (MELISSA) (39) was utilized for the offline mass separation of ^169^Er. ^169^Er-containing ampoules were opened and transferred into an ISOLDE standard tantalum target container (25). The container was connected to a rhenium ion source *via* a transfer line. The extraction electrode was positioned after an acceleration gap of 60 mm from the ion source's exit. Respective currents of 250 A and 300 A were applied to heat the target, as well as the line, and to allow for preliminary optimization steps on stable ^168^Er. ^169^Er was extracted from targets that were heated up to 2,200°C (corresponding to a current of 730 A). The current laser setup consists of two Z-cavity Ti:sapphire lasers, designed by Mainz University (47), each pumped by a dedicated commercial InnoLas Nanio 532-18-Y laser system. The two-step laser resonance ionization scheme for erbium was used; the optimized wavelengths during the collections were 24943.95 cm^−1^ for TiSa n°2 and 24337.32 cm^−1^ for TiSa n°1, as reported in (37, 44, 48). The ions were electrostatically accelerated to 60 keV and mass-separated with a magnetic sector field.

The mass-separated *A* = 169 beam, containing ^169^Er, was implanted into a solid catcher (zinc-coated gold foil). Gold foils (thickness: 0.1 mm, purity: 99.95%, Goodfellow Cambridge Ltd. Huntingdon, UK) were coated with 500 nm Zn (99.995% Zn granulate from Neyco, France) layer using physical vapor deposition (PVD) technique. In total, seven mass-separated samples were shipped to PSI for chemical separation, quality control, and an *in vitro* proof-of-concept experiment.

### Radiochemical Separation

The radiochemical separation method was developed for the separation of macro amounts of Zn and as well as isobaric ^169^Yb and ^169^Tm from the desired ^169^Er. The gold foil obtained from CERN (Supplementary Figure 1) was introduced into a reaction vial and the ^169^Er-implanted Zn layer dissolved in 7 mL 6 M HNO_3._ The resulting solution was directly loaded onto a column containing N,N,N′,N′-tetra-n-octyldiglycolamide, non-branched resin (DGA, particle size 50–100 μm, TrisKem International, France; volume 0.08 mL), which is based on tetraoctlyldigycolamide as sorbent. The column was rinsed several times with 6.0 M HNO_3_ and the Zn concentration in each fraction determined using an Agilent 5110 Inductively Coupled Plasma Optical Emission Spectrometry (ICP-OES).

After removal of the macro amounts of Zn; Er, Yb, and Tm were eluted using 0.05 M HCl and loaded onto a column containing Sykam macroporous cation exchange resin (Sykam Chromatographie Vertriebs GmbH, Germany; particle size 12–22 μm, ${\text{NH}}_{4}^{+}$ form; column volume: 2.5 mL). Separation of ^169^Yb and ^169^Er was performed with 0.06–0.08 M α-hydroxyisobutyric acid (α-HIBA, Sigma-Aldrich GmbH, Germany) using the Sykam cation exchange resin separation system. Subsequently, LN3 resin (Triskem International, France; column volume: 0.04 mL) was used to remove the complexing agent (α-HIBA), be an additional means of Zn removal and to obtain the final product in chloride form. The use of 2 mL 0.02 M HCl removed the Zn from the system, prior to the elution of ^169^Er (1 mL 2.0 M HCl).

As an extra, and final, separation step, ^169^Er 1 mL 2 M HCl solution was passed through a TK200 resin [Triskem International, France; based on TriOctylPhosphine Oxide (TOPO), column volume: 0.06 mL], for the complete removal of Zn from the final product. The eluant was, then, heated until dryness and redissolved in 250 μL 0.05 M HCl solution, which was used for the radiolabeling of PSMA-617.

### Quality Control

#### Activity Standardization

Gamma-ray spectra of the gold foils were taken before and after leaching of the Zn layer, using a high-purity germanium (HPGe) detector (Canberra, France), in combination with the Inter-Winner software package (version 7.1, Itech Instruments, France) (Supplementary Figure 2).

**Figure 2:**
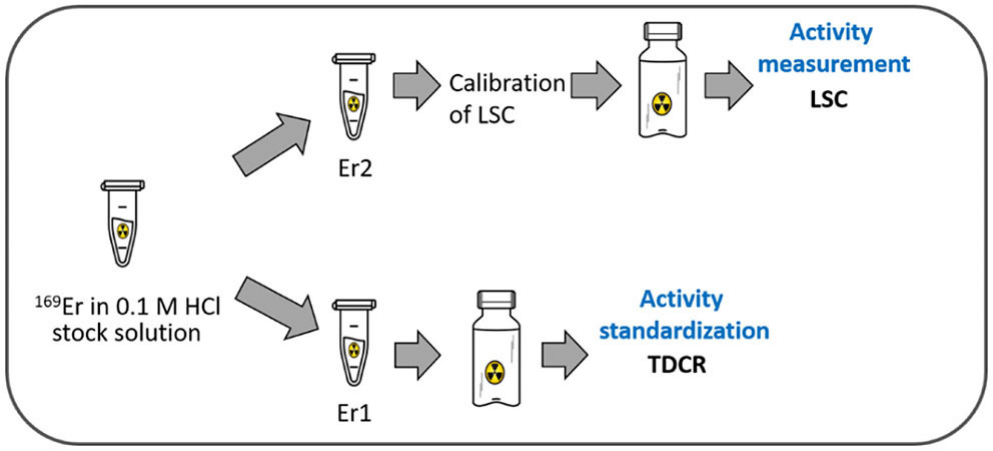
Scheme of the samples used for the activity standardization and calibration of the LSC (liquid scintillation counting).

Purified ^169^Er in 0.1 M HCl (stock solution) was divided into two parts, named Er1 and Er2, respectively (Figure 2). They were accurately weighed using a Mettler-Toledo XS225DU balance. Sample Er1 was sent to the Institute of Radiation Physics (IRA, Lausanne) for activity standardization using the TDCR technique. The activity concentration of the Er1 solution was used to prepare ^169^Er quench series to calibrate LSC using Er2 solution (Supplementary Figure 3A). The counting efficiency for typical samples was ~97%.

The ^169^Er activity measurements of the mass-separated samples were performed after chemical separation using liquid scintillation counting (LSC; LSC Packard Tri-Carb 2250 CA) (Supplementary Figure 3B). Further details about activity standardization measurements of ^169^Er are described in the Supplementary Material.

#### Radionuclidic Purity

After chemical separation, the radionuclidic purity of the samples was determined using a HPGe detector, as described in section Activity Standardization.

#### Isotopic Ratio Measurement

Enriched ^168^Er_2_O_3_ (98.0%, ISOFLEX, USA, and 98.2% Trace Sciences Int. Canada) and four mass-separated ^169^Er samples (S1-S4) were analyzed using a Thermo Fisher Scientific Sector Field Inductively Coupled Plasma Mass Spectrometer (SF-ICP-MS) Element II®. The measurement solutions were prepared from Suprapur nitric acid (Merck GmbH, Germany) and Milli-Q-water, which were also measured and subtracted as blank. Rhenium standard solution (10 mg/L, ESI, Elemental Scientific, Omaha, NE, USA) was added to each measurement solution as an internal standard, to account for plasma instabilities during the measurement. Instrument settings, such as gas flows, were tuned daily.

The measurement was performed in low-resolution mode, scanning the masses 149–170 and for the internal standard 185 and 187. A standard solution containing stable isotopes of rare earth elements, including Er, was measured at the start of the measurement sequence as a performance check.

#### Chemical Purity

The chemical purity of the sample was determined using ICP-OES (Agilent ICP-OES 5110). Zn- and Er-containing standard solutions (0.1, 0.5, 1, 5 ppm, respectively) were prepared in 2% HNO_3_ (Merck Suprapur), using Sigma-Aldrich TraceCERT®, 1,000 mg/L Er and Zn ICP standards.

#### Radiochemical Purity

Radiochemical purities of Sample 5 and Sample [6-7 (dissolved together to obtain higher activity)] were determined by radiolabeling, using PSMA-617 as a model compound. The percentage of ^169^Er-PSMA-617 obtained was determined using High-Performance Liquid Chromatography (HPLC) with a C-18 reversed-phase column (Xterra^TM^ MS, C18, 5 μm, 150 × 4.6 mm; Waters). The mobile phase consisted of MilliQ water containing 0.1% trifluoracetic acid (A) and acetonitrile (B) with a gradient of 95% A and 5% B to 20% A and 80% B over a period of 15 min at a flow rate of 1.0 mL/min. A sample of the radiolabeling mixture was diluted in MilliQ water containing sodium diethylenetriamine-pentaacetic acid (Na-DTPA; 50 μM) prior to injection into the HPLC instrument.

##### System evaluation

^169^Er has a negligible emission of photon radiation. Therefore, before labeling experiments, the detection of ^169^Er using a radio-HPLC detector was assessed by comparison to ^177^Lu. Supplementary Figure 4 shows the comparison of the HPLC chromatograms of ~0.3 MBq free activity for ^169^Er and ^177^Lu, respectively. Before injecting the samples, the HPLC column was cleaned by running several blanks using DTPA solution to ensure the detection of ^169^Er.

##### Preparation of radioligands

PSMA-617 (ABX GmbH, Radeberg, Germany) was labeled with ^169^Er under standard conditions at a molar activity of 10 MBq/nmol. A stock solution of PSMA-617 (1 mM) was mixed with a solution of ^169^Er in 0.05 M HCl and the pH adjusted to 4.0 with the addition of sodium acetate solution (0.5 M, pH 8). The reaction mixture was incubated for 20 min at 95°C. Quality control of ^169^Er-PSMA-617 was performed using HPLC, as reported above.

As a control compound, PSMA-617 was labeled with ^177^Lu (carrier-free in 0.04 M HCl; ITM, Germany) at a molar activity of 10 MBq/nmol, according to a previously-published procedure (49). Quality control of ^177^Lu-PSMA-617 was also performed using HPLC.

### *In vitro* Viability Studies

#### Cell Culture

Sublines of the androgen-independent PC-3 human prostate cancer cell line, PSMA-positive, PC-3 PIP tumor cells were kindly provided by Prof. Martin Pomper (Johns Hopkins University School of Medicine, Baltimore, MD, USA) (50). The cells were grown in RPMI cell culture medium supplemented with 10% fetal calf serum, L-glutamine and antibiotics, as well as puromycin (2 μg/mL), to maintain PSMA expression (51–53).

A tumor cell viability assay was performed using PC-3 PIP/flu tumor cells treated with ^169^Er-PSMA-617 and ^177^Lu-PSMA-617 (0.01–10 MBq/mL), respectively. The assay was performed according to a previously-published method using a 3-(4,5-dimethylthiazol-2-yl)-2,5-diphenyltetrazolium bromide (MTT) assay (7). PC-3 PIP tumor cells (2,500 cells in 200 μL RPMI medium with supplements) were seeded in 96-well plates and incubated overnight (37°C; 5% CO_2_) to allow adhesion of the tumor cells. ^169^Er-PSMA-617 or ^177^Lu-PSMA-617 (10 MBq/nmol, the highest achievable molar activity for ^169^Er-PSMA-617) were diluted in RPMI medium without supplements to an activity concentration of 0.1–10 MBq/mL. The tumor cells were washed with 200 μL phosphate buffered saline (PBS, pH 7.2) prior to the addition of the radioligand dilutions (200 μL/well). Control cells were sham-treated with RPMI medium without the addition of radioligands. After a 4 h incubation period (37°C; 5% CO_2_), tumor cells were washed with 200 μL PBS and fresh PRMI medium (with supplements) was added to each well. Tumor cell viability was determined after 6 days of incubation at 37°C, 5% CO_2_. MTT reagent (5 mg/mL in PBS, 30 μL per well) was added to each well, followed by incubation of the well plates for 4 h. The dark-violet formazan crystals were dissolved with 200 μL dimethyl sulfoxide. The absorbance was measured at 560 nm using a microplate reader (Victor X3, Perkin Elmer). Cell viability was quantified by expressing the absorbance of the test samples as a percentage of the absorbance of untreated tumor cell samples, which was set to 100%.

## Results

### Irradiation and Mass Separation

In total, five ampoules containing ^168^Er_2_O_3_ were used for ^169^Er production at the ILL nuclear reactor, with a theoretical yield between 25 and 48 GBq. After the irradiations, the ampoules were transported to the CERN-MEDICIS facility. Supplementary Figure 5A shows the γ-ray spectrum of commercially-available carrier-added ^169^Er (supplied by b.e. Imaging GmbH) as a representative spectrum for post-irradiation. The product had a reported radionuclidic impurity ≤0.38% of the ^169^Er activity, with ^177^Lu (0.02%) the main impurity.

The mass 169 ion beam was implanted into seven solid catchers (two collections were performed for two ampoules). The mass separation was combined with resonant laser ionization to enhance element selectivity for the erbium ionization, as well as the overall separation efficiency. Thus, ^169^Er activities were increased up to a factor of four compared to the surface ionization technique (37). The presence of trace quantities of Yb (<400 ppm) in the target material (^168^Er_2_O_3_) led to the co-production of ^169^Yb (*t*_1/2_ = 32 d) due to the high thermal neutron capture cross-section of ^168^Yb (σ: 2400 b) (10). As a result, after the mass separation process, ^169^Yb (isobar of ^169^Er) was observed as a radionuclidic impurity (Supplementary Figure 5B). The average collection time was 63 hours, with separation efficiencies of up to 0.5%. To the best of our knowledge, to date, only a few measurements were reported for the emission probabilities of ^169^Er, with large discrepancies (20%) for the two lines at 109.8 and 118.2 keV (54). Moreover, ^169^Er has the same γ-ray peak (109.78 keV, 0.0013%) as ^169^Yb (109.78 keV, 17.39%) with much lower intensity (8), which made difficult to perform precise ^169^Er activity measurements of the samples by means of γ-ray spectrometry. Therefore, separation efficiencies were determined based on the theoretical initial and final ^169^Er activities measured using LSC (after chemical separation). Since activity loss during the ampoule opening and transferring were not taken into consideration, the actual separation efficiencies are expected to be higher than the reported values.

### Radiochemical Separation

**Figure 3:**
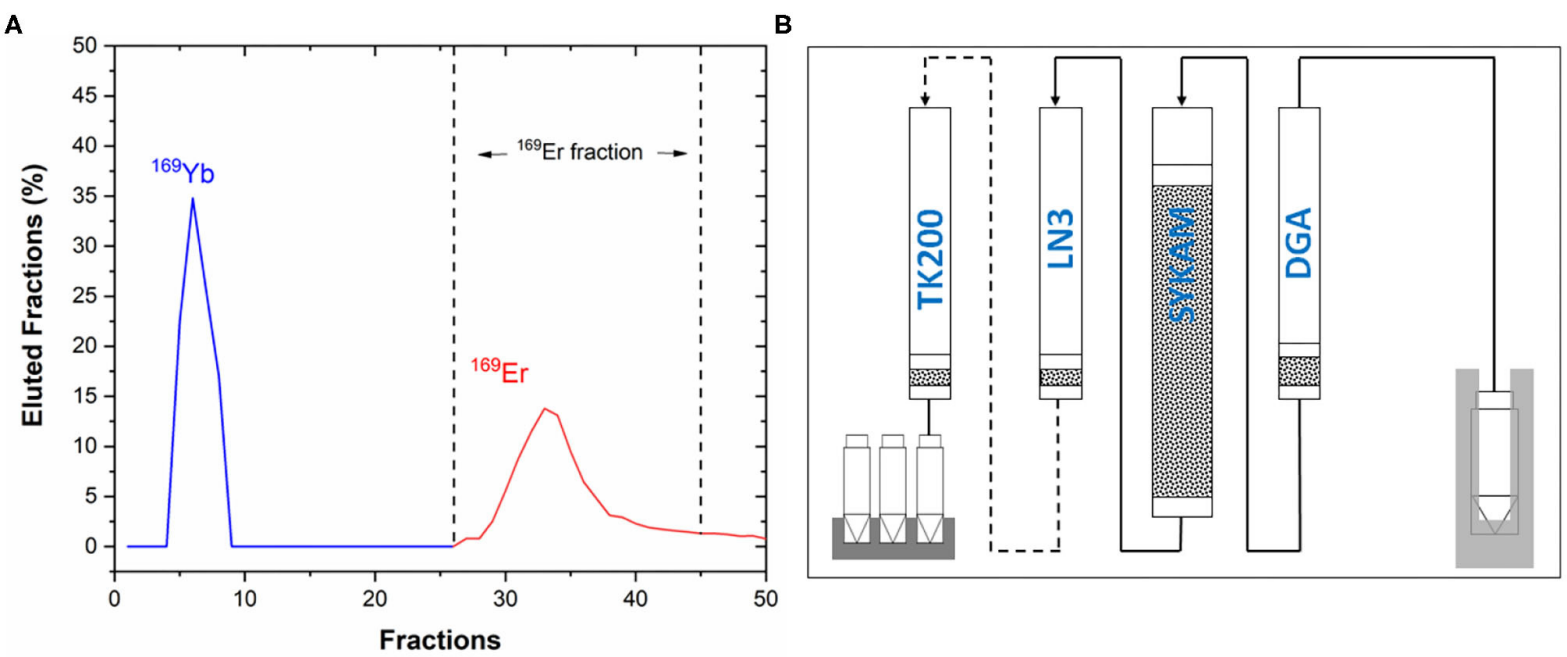
**(A)** Separation profile of ^169^Yb and ^169^Er using Sykam cation exchange resin and α-HIBA as eluent, **(B)** scheme of the chemical separation process for the mass-separated ^169^Er samples.

The zinc layer, containing the implanted ^169^Er activity and the isobar ^169^Yb, was dissolved from the gold foil. Cation and extraction chromatographic resins were used for the effective separation of ^169^Er from isobars and macro quantities of zinc to obtain chemically and radionuclidically pure ^169^Er. DGA extraction resin was used for the first separation step, which required the use of concentrated nitric acid as a loading solution for the separation of macro quantities of Zn from the lanthanides. ICP-OES results showed that traces of zinc (300 ppb) were retained on the resin (Supplementary Figure 6) and eluted together with ^169^Er and ^169^Yb using 0.05 M HCl. The separation of lanthanides (Er and Yb) was, subsequently performed using Sykam cation exchange resin. An example of the separation profile is presented in Figure 3A. The α-hydroxyisobutyric acid (α-HIBA), used as a complexing agent to separate lanthanides, was removed by passing the eluent through LN3 extraction resin. It was also important to use this resin to eliminate Zn from the desired product, as well as to concentrate the desired product in a small volume. This three-step separation process (DGA, Sykam, and LN3 resin) was initially applied to obtain ^169^Er with high radionuclidic and chemical purity. However, quality control analysis of the final product (section Quality Control) showed that a three-step separation process was not sufficient to reach the required high chemical purity. Thus, an additional purification step, using the TK200 resin, was added to separate the remaining Zn from the final product (Figure 3B).

### Quality Control

**Figure 4:**
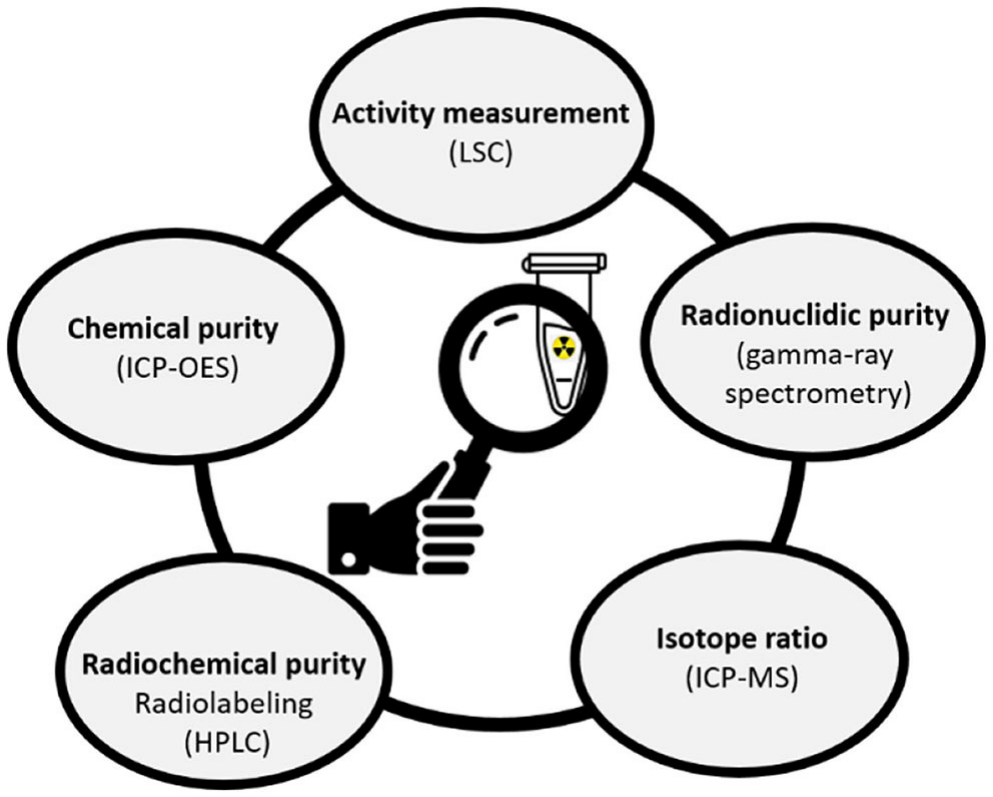
Quality control analyses applied to the ^169^Er samples after chemical separation (LSC, Liquid Scintillation counting, ICP-MS, Inductively Coupled Plasma Mass Spectrometry; HPLC, High-Performance Liquid Chromatography; ICP-OES, Inductively Coupled Plasma—Optical Emission Spectrometry).

Assessment of the quality control steps is crucial for the development of novel radionuclides toward nuclear medical applications (55). As part of the quality control processes, activity, radionuclidic purity, isotope ratio, radiochemical purity and chemical purity measurements were performed to evaluate the success of the mass and chemical separation methods (Figure 4). In total, seven ^169^Er samples were analyzed using different techniques (Table 3). Samples 6 and 7 were dissolved together to obtain higher activity toward an *in-vitro* experiment.

**Table 3:** Quality control analysis and results obtained for the mass-separated samples post-chemical processing.

	**S1**	**S2**	**S3**	**S4**	**S5**	**S6-7**
Activity measurement (MBq)	52.9	23.4	8.59	4.70	73.2	93.4
Radionuclidic purity (%)	>99.9	>99.9	>99.9	>99.9	>99.9	>99.9
Isotopic ratio (168/169)	1.66	1.60	14.62	11.94	n.d	n.d
Chemical purity	n.d	n.d	n.d	n.d	0.49 μg Zn	n.d
Radiochemical purity (%)	n.d	n.d	n.d	n.d	0^*^	>98%

*n.d., not determined*.

* *The radiolabeling of PSMA-617 was not possible*.

#### Activity Standardization

Gamma-ray spectrometry measurements were performed before and after dissolving the zinc layer from the gold foils to evaluate the fraction of the ^169^Er implanted into the zinc layer and the gold backing, respectively. Except for the first sample, the ^169^Er activities that remained on the gold foils were negligible.

In order to perform precise ^169^Er activity measurements, a purified ^169^Er solution was used for the activity standardization using the TDCR technique and its activity concentration was measured as 798.5 ± 5.9 kBq/g (0.33%, *k* = 1). The uncertainty budget of the measurement is given in Supplementary Table 1. An efficiency calibration curve was constructed based on the activity concentration determined by the TDCR technique and stored in the LSC software to enable routine activity measurements. Subsequently, activity measurements of all the purified ^169^Er samples were performed using calibrated LSC.

#### Radionuclidic Purity

**Figure 5:**
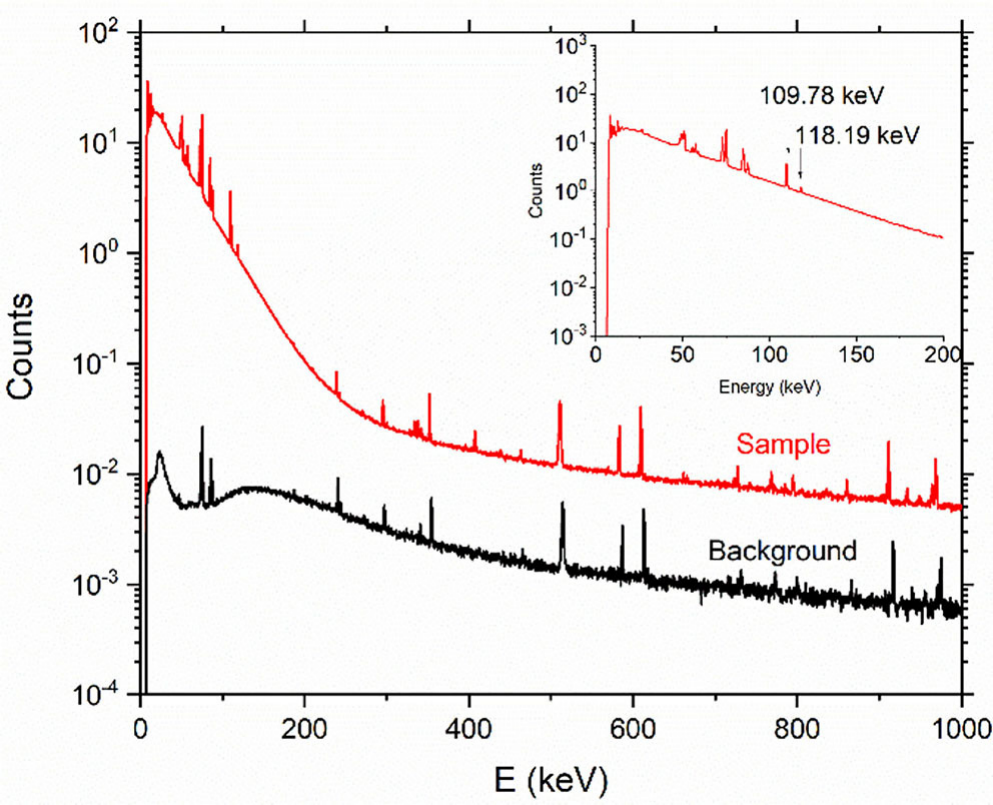
Representative γ-ray spectrum of ^169^Er product after chemical separation (counting time: 15 days, sample detector distance: 15 cm) and background spectrum (counting time: 2 days). The inset shows a zoom of the two lines at 109 and 118 keV, respectively.

Figure 5 shows an example of a γ-ray spectrum of ^169^Er after chemical separation. Thanks to the effective removal of ^169^Yb (Figure 3A), no radionuclidic impurity was determined after 15 days counting time. The background spectrum was taken shortly after the measurement to check for its interference. The two ^169^Er γ-ray peaks at 109 and 118 keV were observed (the inset of Figure 5), as well as the intense peaks at 50 keV and 57–59 keV, which correspond to the X-ray lines of ^169^Er.

#### Isotopic Ratio

After the mass and chemical separation processes, four samples were analyzed to determine the mass 168 and 169 ratios using a Sector Field Inductively Coupled Plasma Mass Spectrometer (SF-ICP-MS). The results showed that the tail of ^168^Er was more pronounced in Samples 3 and 4 than Samples 1 and 2 (Table 3). Sample 5 and Sample 6-7 were not analyzed, since these samples were used for radiolabeling. The analytical method was validated by measuring the ^168^Er enriched materials, which were in good agreement with their certificate of analysis (CoA) (Supplementary Table 2).

#### Chemical and Radiochemical Purity

After the three-step separation process, Sample 5 was used to test the preparation of ^169^Er-PSMA-617 at a molar activity of 10 MBq/nmol. The radiolabeling of PSMA-617, failed due to the presence of 0.49 μg Zn impurity (determined by ICP-OES) competing with the ^169^Er for the DOTA chelator of PSMA-617.

To investigate the quantity of Zn that will affect the labeling efficiency, labeling experiments were performed with non-carrier-added ^177^Lu (as a surrogate for ^169^Er) and DOTANOC in the presence of different masses of non-radioactive Zn (Supplementary Figure 7). These experiments revealed that radiolabeling of the compound was not possible in the presence of ≥0.2 μg Zn. Thus, samples 6 and 7 were dissolved together and purified, utilizing TK200 resin to separate the remaining Zn.

##### Preparation of radioligand

**Figure 6:**
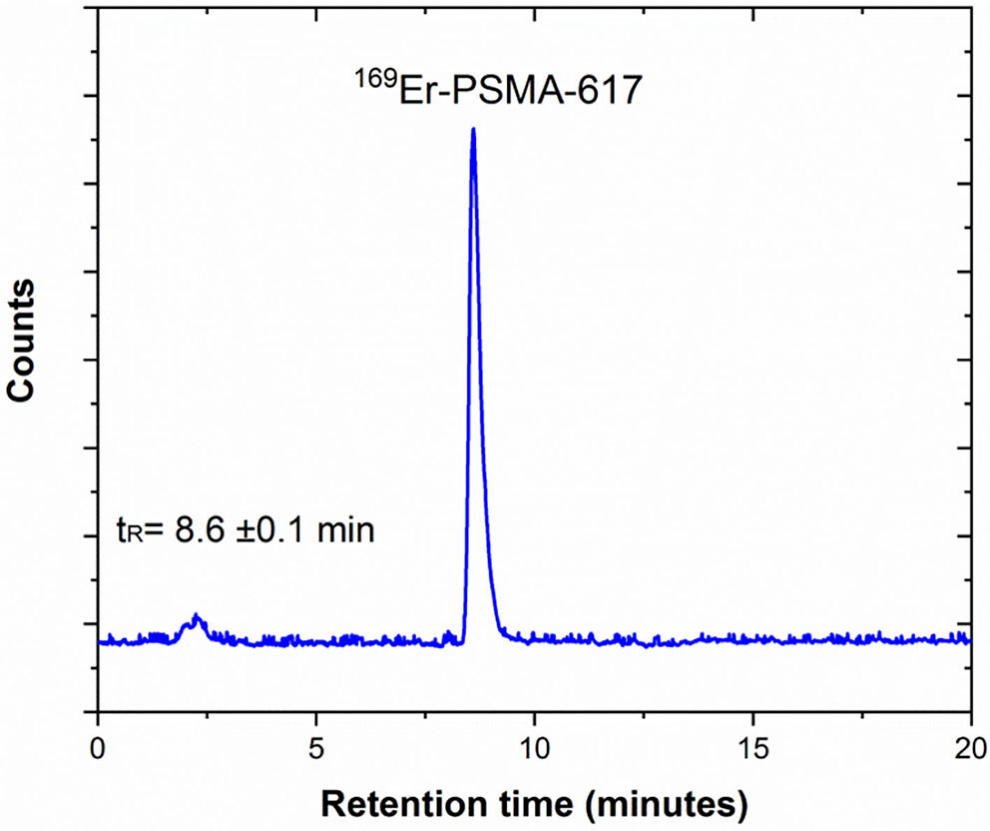
Representative HPLC radiochromatogram of ^169^Er-PSMA-617 prepared at 10 MBq/nmol, demonstrating the product peak and its retention time in minutes (*t*_R_ = 8.6 ± 0.1 min) (pH: 4.5, T: 95°C, 20 min incubation).

Samples 6 and 7 were dissolved, combined, and used to prepare ^169^Er-PSMA-617 at a molar activity of 10 MBq/nmol with a radiochemical purity of >98% (Figure 6).

### *In vitro* Viability Studies

**Figure 7:**
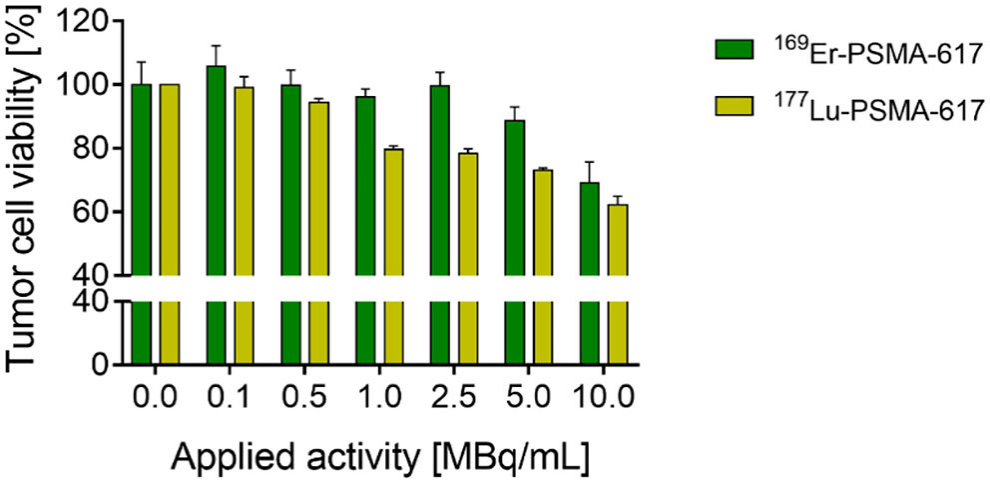
Results of PC-3 PIP tumor cell viability study performed with ^169^Er-PSMA-617 (*n* = 1, SD refer to intraexperimental variation) and ^177^Lu-PSMA-617 (*n* = 3, SD refer to interexperimental variation).

The radioligand ^169^Er-PSMA-617 was tested in a tumor cell viability assay (*in vitro*) to investigate its therapeutic potential in comparison to the clinically-established ^177^Lu-PSMA-617. Exposure of PC-3 PIP tumor cells to ^169^Er-PSMA-617 resulted in reduced tumor cell viability (89 ± 4% and 69 ± 7%) when 5 and 10 MBq/mL, respectively, were applied. At lower concentrations of the applied radioligand (0.1–2.5 MBq/mL) the cell viability was unaffected compared to sham-treated control cells. ^177^Lu-PSMA-617 had a greater effect on tumor cell viability, resulting in 72 ± 1% and 62 ± 3% viable cells after exposure to 5 and 10 MBq/mL, respectively. Also, tumor cell viability was reduced at lower applied activity concentrations of 1.0 and 2.5 MBq/mL resulting in 80 ± 1% and 79 ± 1% viable tumor cells, respectively (Figure 7). It is important to note that, due to the limited availability of ^169^Er, the experiment could only be performed once in a setting that does not fully reflect the therapeutic potential of this novel radionuclide. The molar activity of ^169^Er-PSMA-617 was relatively low (10 MBq/nmol), and hence, receptor saturation effects were likely to occur. In order to enable effective cell killing, the radioligand should be prepared at higher molar activity and applied at higher activity concentrations.

## Discussion

In the previous mass separation experiment at the CERN-MEDICIS facility, ^169^Er was thermally ionized (37). This ionization method, however, was prone to isobaric contamination from elements with similar or lower ionization potential (37). Different studies performed on other radionuclides demonstrated that element-selective laser resonance ionization can provide significantly higher ionization efficiency for the element of interest and, thus, reduce the concentration of isobars in the final product (38, 42). As a result, the surface ionization technique used at MEDICIS was upgraded with a resonant ionization laser ion source. Although higher activities of ^169^Er were extracted compared to the previous study (^169^Er activities were increased up to a factor of four) (37), there is still room for further improvement of the overall efficiency and beam purity by developing an *ad hoc* isotope-selective ionization process for ^169^Er. The quantity of ^168^Er target material is several orders of magnitude greater than the ^169^Er produced; the erbium ionization led to a broad ^168^Er ion beam after the electromagnet, which tailed on neighboring masses, namely, at mass 169. As one can see from Table 3, it resulted in a prevalence of ^168^Er over ^169^Er still after mass separation. A first trial to increase the ^169^Er isotopic ratio, by narrowing laser linewidth, failed, and the results worsened (collected samples S3 and S4). Furthermore, the parallel ionization of bulk amounts of ^168^Er can reduce the ion source performance, impeding the proper separator adjustment. This can be solved with the application of an isotope-selective laser ionization scheme or optimization of the mass separator for high-flux ion-beam operation (40).

During the mass separation process, isobaric contaminants can also be extracted due to surface ionization and non-resonant laser ionization. They could also be collected in the Zn-coated gold foils, such as ^169^Yb, which was observed by γ-ray spectrometry after the mass separation process. The presence of ^169^Yb would affect the dosimetric evaluation of the ^169^Er radiopharmaceutical. Moreover, the chemical purity of the final product would have a direct effect on the radiolabeling of the desired radionuclide to a target molecule (56, 57). Thus, a radiochemical separation process was necessary for the separation of ^169^Er from the isobaric contaminants (^169^Yb) and soluble residues of the Zn-coated gold foil to make the desired radionuclide available in a purified form suitable for radiolabeling procedures.

As mentioned previously, separation of lanthanides is a particularly challenging issue, due to their similar chemical behavior. Previously, Chakravarty et al. used an electrochemical separation method for the complete removal of ^169^Er from ^169^Yb (58). The method was based on a two-step electro-amalgamation technique, the selective reduction of Yb^3+^ to Yb^2+^ followed by its preferential amalgamation on to a mercury cathode. To date, considerable effort has been expended on the testing of various chelating agents for lanthanide separation (59). It was shown that α-hydroxy-isobutyric acid (α-HIBA) exhibits a consistent trend across the entire lanthanide series (60–64). In the present study, the complete removal of ^169^Yb from ^169^Er was accomplished using Sykam macroporous cation exchange resin and α-HIBA eluent. Zinc separation was achieved using different extraction resins (DGA, LN3, and TK200). It is worth mentioning that the Zn quantity reported in the commercially-available non-carrier-added ^177^Lu (ITM, Germany) is ≤0.1 μg/GBq with an activity concentration of 37.5 GBq/mL (80 μL 3 GBq ^177^Lu can contain 0.3 μg Zn). This quantity of Zn does not affect labeling due to the high activity concentration of the ^177^Lu solution. However, the activity concentration of the mass-separated ^169^Er solutions are much lower than the commercially-available non-carrier-added ^177^Lu solution. Thus, even ppb levels of Zn impurity could affect the labeling efficiency (Table 3).

In order to characterize ^169^Er with regard to its potential as a therapeutic radionuclide, extensive *in vitro* and *in vivo* studies will be necessary. In this regard, the stability of DOTA complexes has to be assessed to confirm stable coordination of ^169^Er using biomolecules that are currently used with ^177^Lu. This will, however, only be possible once the radionuclide can be made available in larger quantities and at a quality that allows the preparation of radioligands at high molar activity as it is feasible with other therapeutic radionuclides, including ^177^Lu and ^161^Tb.

A typical clinical dose of ^169^Er (E$\beta_{\text{av}}^{-}$ = 100 keV) is expected to be several GBq, comparable to that of clinically applied ^177^Lu (E$\beta_{\text{av}}^{-}$ = 133.6 keV) labeled PSMA-617. As was presented, the activities obtained were sufficient for a preliminary *in vitro* assay, however, still fall well short for extensive pre-clinical studies. If the overall efficiencies could be improved from 0.5 to 20% (39, 43–45), it will be possible to reach higher ^169^Er activities. Moreover, for the present study, irradiations were performed for 7 days. For an industrial production, one could irradiate for longer, minimizing the activity loss during transport and chemical separation, and increasing the yield of ^169^Er.

## Conclusion

In this study, radionuclidically pure ^169^Er was produced at high specific activities by means of neutron irradiation, followed by mass and chemical separation processes, respectively. By combining surface ionization with resonant laser ionization, ^169^Er activities were increased up to a factor of four compared to the previously-published study. Further developments of the mass separation process, such as the application of an isotope-selective laser ionization scheme and optimization of the mass separator for high-flux ion-beam operation, are needed to increase the overall separation efficiencies and to provide higher activities of ^169^Er in future. The availability of the β^−^-particle-emitting ^169^Er is particularly important to perform pre-clinical studies, in combination with the pure Auger electron emitter ^165^Er. This combination will help us explore the additional therapeutic effect of Auger electrons to β^−^-particles, as in the case of ^161^Tb, which will certainly be of scientific relevance. Enhanced knowledge of the additive therapeutic effects of Auger electrons will likely be well-received by the nuclear medicine community and ought to pave the way toward more efficient cancer treatment, particularly, for the treatment of single cancer cells and small metastases.

## Data Availability Statement

The original contributions presented in the study are included in the article/Supplementary Material, further inquiries can be directed to the corresponding author.

## Author Contributions

All authors listed have made a substantial contribution to the work, read, and agreed to the published version of the manuscript.

## Conflict of Interest

The authors declare that the research was conducted in the absence of any commercial or financial relationships that could be construed as a potential conflict of interest.

## Footnotes

^1^https://www.nndc.bnl.gov/nudat2/ (accessed March 3, 2021).
