# Supplementary material for: Production of Mass-Separated Erbium-169 Towards the First Preclinical in vitro Investigations
Source: Front Med (Lausanne). 2021 Apr 22;8:643175. doi: 10.3389/fmed.2021.643175 (PMC8100037; doi:10.3389/fmed.2021.643175)
Supplement: Supplementary file 1 [file Data_Sheet_1.docx]

**Supplementary Material**

**Production of Mass Separated Erbium-169 Towards the First Preclinical in-vitro investigations**

Z. Talip^1*^, F. Borgna^1^, C. Müller^1,2^, J. Ulrich^3^, C. Duchemin^4,5^, J. P. Ramos^4,5^, T. Stora^4^, U. Köster^6^, Y. Nedjadi^7^, V. Gadelshin^4,8,9^, V. N. Fedosseev^4^, F. Juget^7^, C. Bailat^7^, A. Fankhauser^10^, S. G. Wilkins^4^, L. Lambert^4^, B. Marsh^4^, D. Fedorov^11^, E. Chevallay^4^, P. Fernier^4^, R. Schibli^1,2^, N. P. van der Meulen^1,3^

^1^Center for Radiopharmaceutical Sciences ETH-PSI-USZ, Paul Scherrer Institute, 5232 Villigen-PSI, Switzerland

^2^Department of Chemistry and Applied Biosciences, ETH Zurich, 8093 Zurich, Switzerland

^3^Laboratory of Radiochemistry, Paul Scherrer Institute, 5232 Villigen-PSI, Switzerland

^4^CERN, 1211 Geneva, Switzerland

^5^Institute for nuclear and radiation physics, KU Leuven, 3001 Leuven, Belgium

^6^Institut Laue-Langevin, 38042, Grenoble, France

^7^Institute of Radiation Physics, University Hospital and University of Lausanne, 1007 Lausanne, Switzerland

^8^Institute of Physics, Johannes Gutenberg University, 55128 Mainz, Germany

^9^Institute of Physics and Technology, Ural Federal University, 620002 Yekaterinburg, Russia

^10^Analytic Radioactive Materials, Paul Scherrer Institute, 5232 Villigen-PSI, Switzerland

^11^ Petersburg Nuclear Physics Institute, NRC Kurchatov Institute, Gatchina 188300, Russia

***Corresponding author**:

Dr. Zeynep Talip

Center for Radiopharmaceutical Sciences ETH/PSI/USZ

Paul Scherrer Institut

5232 Villigen-PSI

Switzerland

e-mail: zeynep.talip@psi.ch

phone: +41-56-310 2486

**Figure S1.** An example of ^169^Er-implanted Zn coated gold foil image before (a) and after (b) dissolving the Zn layer.

**Figure S2.** Examples of gamma-ray spectra of the gold foils before (sample 1 (a), sample 2 (c)) and after dissolving the Zn layer (sample 1 (b), sample 2 (d)) (measurements were performed with the same sample detector distance and counting time).

**Figure S3.** The plot of the measured efficiencies used for the preparation of the ^169^Er calibration curve (a) and LSC spectrum of ^169^Er (b).

**Figure S4.** Comparison of the free (uncomplexed) 0.27 MBq ^169^Er and 0.3 MBq ^177^Lu HPLC radiochromatograms.

**Figure S5**. Examples of gamma-ray spectra of (a) carrier-added ^169^Er (supplied from b.e. Imaging GmbH and stated as a representative spectrum of post-irradiation, indicating what can be expected and (b) mass-separated ^169^Er.

**Figure S6.** An example of DGA column study for the separation of Zn (column volume: 0.08 mL, loading solution: 7 mL 6 M HNO_3_; F (fraction): 6 mL 6 M HNO_3_).

**Figure S7.** Comparison of the HPLC radiochromatograms of ^177^Lu-DOTANOC contains additional 2 µg (a), 0.2 µg (b) and 0.02 µg (c) Zn (labelling conditions: pH: 4.5, T: 95^o^C, 10 minutes incubation for 100 MBq/nmol).

**Table S1.** Uncertainty budget for the ^169^Er activity measurement.

**Table S2.** Comparison of the measured and CoA mass distribution values for ISOFLEX (98.0% ISOFLEX, USA) and TRACE ^168^Er enriched Er_2_O_3_ (98.2% Trace Sciences Int. Canada).

**Gamma-ray spectrometry measurements of the gold foils**

**Purpose:** Gamma-ray spectrometry measurements of the gold foils to determine the ^169^Er fraction implanted into the Zn layer and gold backing.

**Method:** After mass separation gamma-ray spectrometry measurements of the gold foils (Figure S1a) were performed before and after dissolving the Zn layer (Figure S1b) using high-purity germanium (HPGe) detector (Canberra, France), in combination with the Inter-Winner software package (version 7.1, Itech Instruments, France).

**Result:** It was observed that, except for the first sample (S1), the remaining ^169^Er activities on the gold foils were negligible (Figure S2). This could be due to the too focused beam used for the first collection, thereby, embedding some of the activity into the gold foil.


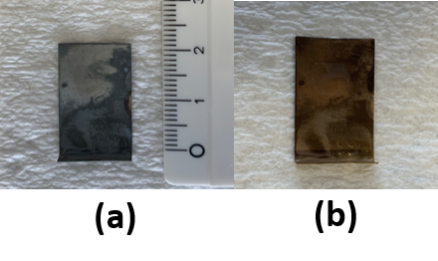


**Figure S1.** An example of ^169^Er-implanted Zn coated gold foil image before (a) and after (b) dissolving the Zn layer.


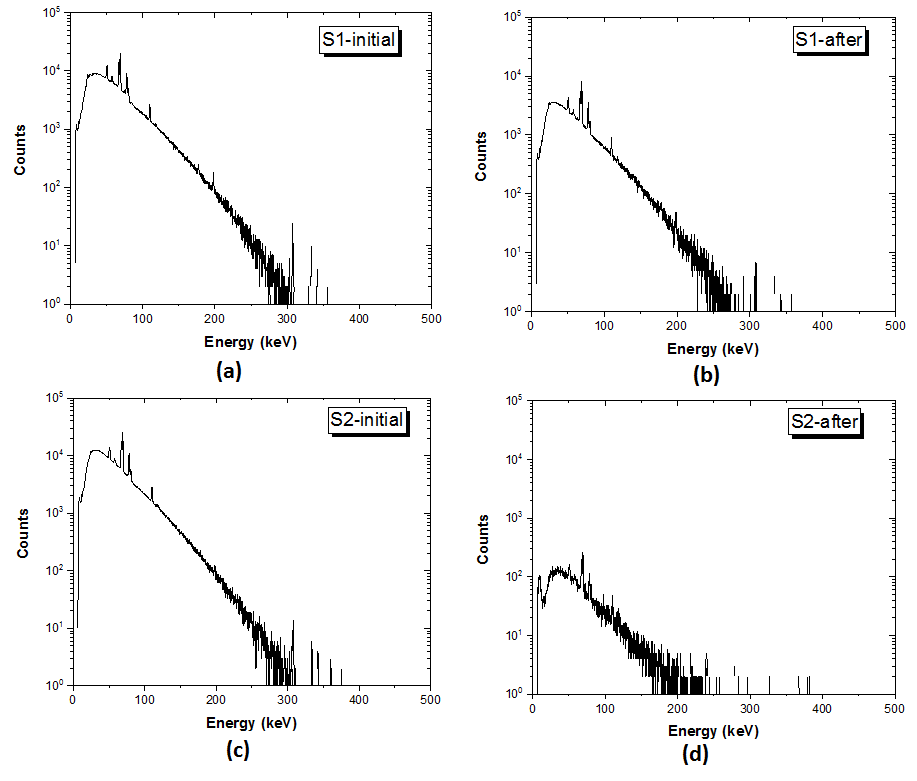


**Figure S2.** Examples of gamma-ray spectra of the gold foils before (sample 1 (a), sample 2 (c)) and after dissolving the Zn layer (sample 1 (b), sample 2 (d)) (measurements were performed with the same sample detector distance and counting time).

**Erbium-169 activity measurement**

**Purpose:** Calibration of liquid scintillation counter (LSC) for routine activity measurement of ^169^Er.

**Method:** An aliquot of the ^169^Er solution, which was sent for activity standardization to IRA, was diluted with 0.1 M HCl to an activity concentration of 90.30 kBq/g (reference date 08. July 2019, 12:00:00 UTC). A series of 10 calibration samples with increasing quenching level was prepared by mixing 10 mL Ultima Gold scintillator cocktail (Perkin Elmer), 950 µL 0.1 M HCl and 50 µL of the diluted ^169^Er solution (corresponding to 2 kBq at the time of the measurement) in a polyethylene LSC vial. The exact amount of the ^169^Er solution added to each vial was traced gravimetrically.  Nitromethane was used as a quenching agent, with its content ranging from 0 (quenching value (tSIE) ~550) to 150 µL (tSIE ~50) over the calibration series. A blank was prepared by mixing 10 mL Ultima Gold and 1 mL 0.1 M HCl.

The measurements were carried out on a Packard TriCarb 2250CA liquid scintillation counter.  Background of the blank sample was negligible (30 CPM) compared to the count-rates of the calibration samples (80-120 kCPM). The counting uncertainty of each calibration sample measurement was kept below 0.15% (*k*=1).

**Result:** An efficiency calibration curve was constructed using the determined counting efficiencies (Figure S3a) and stored in the LSC software in order to enable routine activity measurements. It has to be noted that the typical samples showed no to very limited quenching and hence, the typical counting efficiency during these measurements was about 97%. Figure S3b shows an example of the ^169^Er LSC spectrum.

**
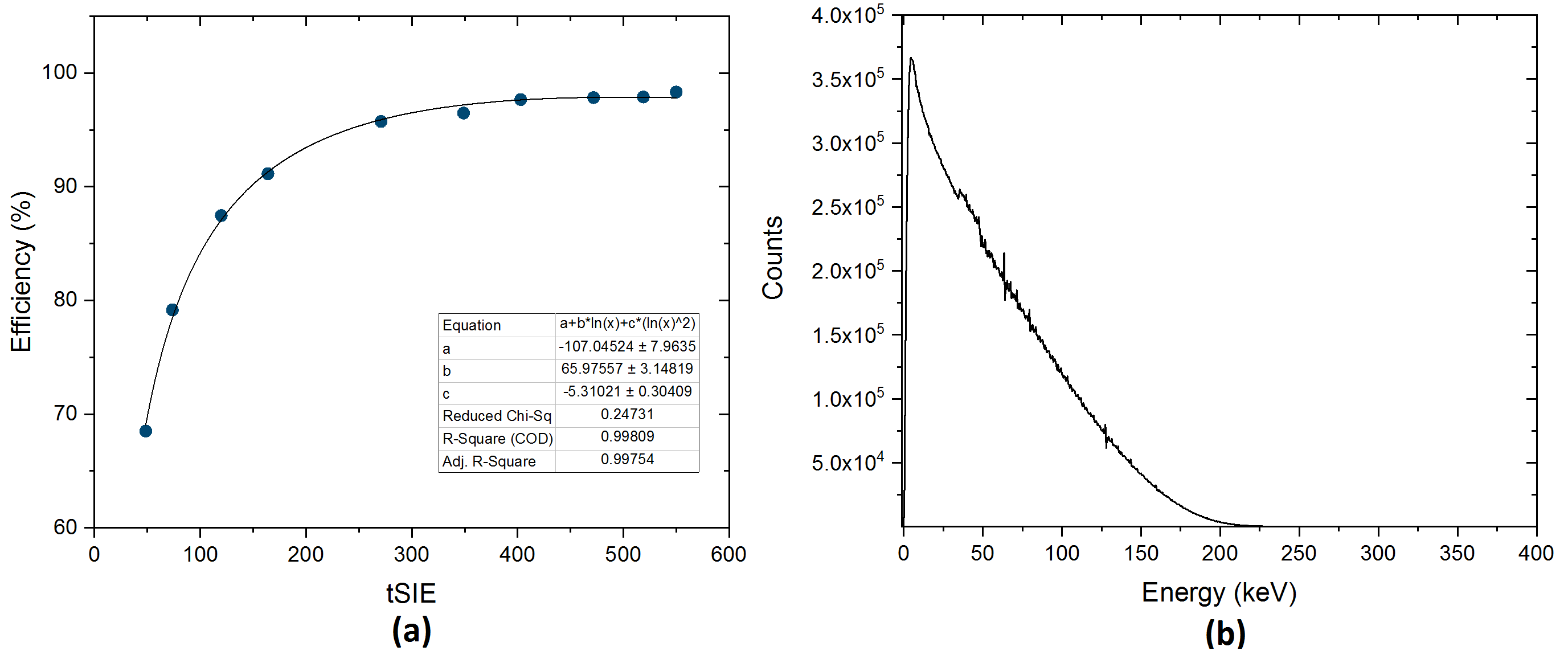
**

**Figure S3.** The plot of the measured efficiencies used for the preparation of the ^169^Er calibration curve (a) and an example of the ^169^Er LSC spectrum (b).

**Radiochromatogram of Erbium-169**

**Purpose:** The detection of ^169^Er was evaluated in comparison to ^177^Lu using a Radio-High-Performance Liquid Chromatography (HPLC) detector.

**Method:** Uncomplexed 0.27 MBq ^169^Er, chemically separated as part of this work, and 0.30 MBq ^177^Lu solutions (supplied from ITM, Germany) was injected into the HPLC system to evaluate the detection of ^169^Er using a radio-HPLC detector.

**Result:** Figure S4 shows a comparison of the HPLC chromatograms of ~0.3 MBq free activity of ^169^Er and ^177^Lu. Before each analysis, proper cleaning of the HPLC column was required due to the low intensity of the ^169^Er peak.

**
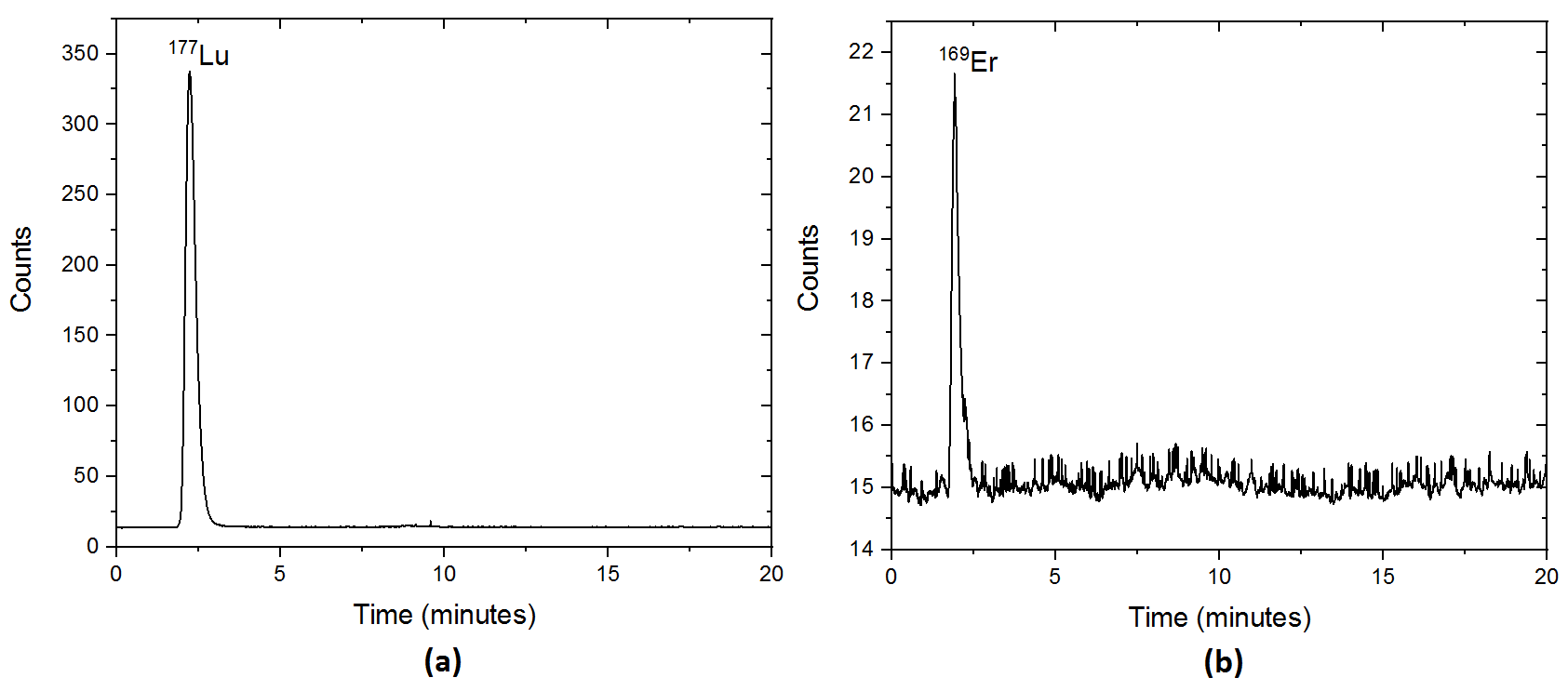
**

**Figure S4.** Comparison of the free (uncomplexed) 0.27 MBq ^169^Er and 0.30 MBq ^177^Lu HPLC radiochromatograms.

**Gamma-ray spectrometry measurement of neutron-irradiated Erbium-168 sample before and after mass separation**

**Purpose:** Comparison of the gamma spectrum of the neutron-irradiated ^168^Er samples before and after mass separation

**Method:** High-purity germanium (HPGe) detector (Canberra, France), in combination with the Inter-Winner software package (version 7.1, Itech Instruments, France) was used for γ-ray spectrometry measurements. The carrier-added ^169^Er (37 MBq), was supplied as a colloidal suspension of ^169^Er citrate from Curium (Swiss distributor b.e. imaging GmbH). It was used as a representative spectrum of post-irradiation.

**Result:** Besides ^169^Er; ^169^Yb, ^175^Yb, ^170^Tm, ^172^Lu, and ^177^Lu gamma-ray peaks were observed in the carrier added ^169^Er solution. Its main impurity was ^177^Lu (0.02%) (Figure S5a). After mass separation only ^169^Er and its isobar ^169^Yb were detected in the γ-ray spectrum.


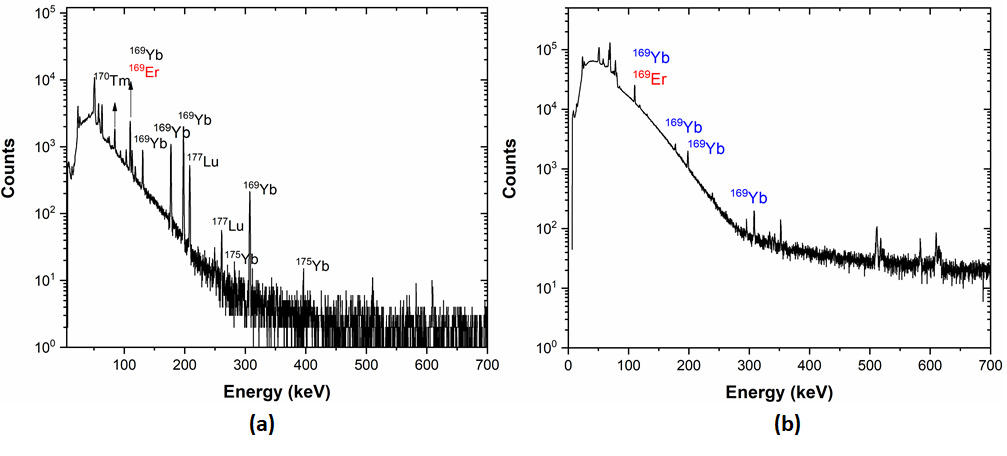


**Figure S5**. Examples of gamma-ray spectra of (a) carrier-added ^169^Er (supplied from b.e. Imaging GmbH and stated as a representative spectrum of post-irradiation, indicating what can be expected and (b) mass-separated ^169^Er.

**Separation of zinc using DGA resin**

**Purpose:** Separation of macro amount of Zn from acidic solution.

**Method:** After mass separation, gold foil was introduced into a reaction vial and the ^169^Er-implanted Zn layer dissolved in 7 mL 6 M HNO_3._ The resulting solution was directly loaded onto a TRISKEM-TODGA (DGA) resin (column`s volume: 0.08 mL), which is based on tetraoctyldigycolamide as sorbent. The column was rinsed several times with 6 M HNO_3_ and sample fractions (6 mL) were collected. The Zn concentration in each fraction was determined using Agilent 5110 Inductively Coupled Plasma Optical Emission Spectrometry (ICP-OES).

**Result:** DGA extraction resin was allowed the use of concentrated HNO_3_ as a loading solution for the separation of macro amounts of Zn. ICP-OES results, however, showed that traces of Zn were retained on the resin.

**

**

**Figure S6.** An example of DGA column study for the separation of Zn (column volume: 0.08 mL, loading solution: 7 mL 6 M HNO_3_; F (fraction): 6 mL 6 M HNO_3_).

**The effect of zinc impurity on labeling efficiency**

**Purpose:** Test experiments were performed with no-carrier-added ^177^Lu (supplied from ITM, Germany) and DOTANOC with the presence of different amounts of Zn to investigate the Zn impurity effect on labeling.

**Method:** Zn solutions, containing 2, 0.2, and 0.02 µg Zn in 0.05 M HCl, were prepared using Sigmaldrich ICP standard TraceCERT^®^, 1000 mg/L Zn ICP standards. These solutions were, then, used for radiolabeling of DOTANOC (ABX GmbH, Germany) at a molar activity of 100 MBq/nmol with ^177^Lu. The labeling was performed at pH 4.5, 95 ^°^C, and 10 minutes incubation time.

**Result:** It was shown that the formation of ^177^Lu-DOTANOC is highly dependent on the concentration of Zn (Figure S7). Radiolabeling of DOTANOC was not possible in the presence of 0.2 and 2 µg Zn.





**Figure S7.** Comparison of the HPLC radiochromatograms of ^177^Lu-DOTANOC containing additionally 2 µg (a), 0.2 µg (b) and 0.02 µg (c) of Zn (labeling conditions: pH 4.5, T: 95 ^°^C, 10 minutes incubation for 100 MBq/nmol).

**Erbium-169 activity standardization**

**Purpose:** After chemical separation, an aliquot of the radionuclidically pure ^169^Er solution in 0.1 M HCl was sent to the Institute of Radiation Physics (IRA, Lausanne) for activity standardization using the triple to double coincidence ratio (TDCR) technique.

**Method:** The TDCR method is a primary measurement technique for standardizing the activity of beta-emitting and electron capture radionuclides (1,2). IRA’s TDCR electronic system was adjusted for an optimal response for ^169^Er (3). The thresholds were set at the valleys of the single-electron responses of the three PMTs. A study of the variation of the double and triple coincidence rates with the coincidence window was made to optimize the resolving time. Sets of ^169^Er sources were prepared by dispensing gravimetrically, with a pycnometer, the ^169^Er solution into 20 mL low-potassium high-performance sandblasted glass vials prefilled with 14.5 mL Ultima Gold cocktail. Each sample was agitated for 2 minutes with a vortex shaker and then centrifuged at 15 revolutions per second for 150 s to settle down the liquid on the cap and walls. After 24 hours in the dark, the samples were measured on the TDCR counter using three efficiency variation methods: defocusing the photomultiplier tubes by varying from the focus voltage between 560 and 320 V in 20 V decrements, optical filtering using plastic stickers of variable optical densities around vials, and by quenching with multiple volumes of carbon tetrachloride. The efficiencies and activities were calculated using IRA’s code which takes the PMTs asymmetry and the micelle effect into account (4). This code computes the efficiencies using the stochastic approach, in which the multimillion sampled energies of the beta particles, the electrons, and photoelectrons produced in the vial are obtained from Geant 4 Monte Carlo simulations involving the Radioactivity and Atomic relaxation modules. Nuclear and atomic data used are from Nucleide-LARA and ENSDF (5,6).

The full uncertainty budget is given in Table S1. The counting statistics component was estimated using a defocusing measurement with 12 counting points (560 to 340 V in 20 V steps). 10^3^ quintuplets of correlated random Gaussian net coincidence counting rates (R_AB_, R_BC_, R_AC_, R_D_, R_T_) were generated for each of their counting points. These sets of Monte Carlo simulated rates were generated in such a way as to reproduce the corresponding experimental moments and covariance matrices. The 10^3^ grey filtering data sets and 10^3^ defocusing data sets were then fed into the code which computes the efficiencies and activities. The ESTAR stopping powers were used and the ionization quenching parameter (kB) was fixed at 0.012 cm/MeV (7). The standard deviation of the distribution of the activities thus obtained was assumed to be the propagation of the rates’ probability density functions on the activity.

The background component was estimated using the same defocusing measurement discussed above. 10^3^ sets of correlated random Gaussian background counting rates (B_AB_, B_BC_, B_AC_, B_D_, B_T_) were generated for each counting point. The simulated rates have mean values, standard deviations of the means, and covariance matrices in agreement with the measured background rates. Corresponding net counting rates were generated by subtracting the Monte Carlo background rates from the measured gross counting rates. The same procedure as above was used to compute the propagation of the background uncertainties on the activity. The relative standard uncertainty was found to be 0.006 %.

For the decay scheme contribution, the uncertainties of the intensities of the main beta branches 44(5)% and 56(5) % were focused, respectively. As a result, the activity of the sample was determined using defocusing, with 49% and 51% beta intensities, in one case, and with 39 and 61% beta intensities in the second case. The relative standard deviation was calculated assuming a rectangular distribution.

Concerning the propagation of the uncertainties of the ionization quenching function and ionization quenching parameter, the procedure used was as follows. Separate sets of 2×10^3^ uniform deviate of kB lying between 0.008 and 0.0120 cm/MeV were generated, assuming a rectangular distribution, and then coupled with five ionization quenching function to compute altogether 10000 activities. Four of these ionization quenching functions were obtained from the ESTAR stopping powers, and those of Gümüs (8) and Tan and Xia (9), and the fifth is that of Grau Carles and Grau Malonda (10). This procedure was performed for the defocusing measurement discussed above. The relative standard deviation of the 10^4^ activities thus generated is 0.097 %. As regards the weighing component, the relative standard of the lightest source inset was taken. The largest component is the relative standard deviation of the mean activity of all the sources used with the 3 efficiency variation techniques (defocusing, optical filtering, quenching).

**Result:** The activity concentration of the ^169^Er solution was measured to be 1798.53 ± 5.93 kBq/g, (0.33 %, *k*=1) on 8.7.19 at 12 h UTC. It is the arithmetic average of measurements performed with the defocusing, optical filtering, and quenching methods.

The full uncertainty budget of the measurement is given in Table S1. The total uncertainties were calculated by combining individual uncertainty contributions (by taking the square sum of all uncertainty components).

**Table S1.** Uncertainty budget for the ^169^Er activity measurement

| Counting statistics | 0.120% |
| --- | --- |
| Background | 0.006% |
| Decay scheme | 0.182% |
| kB and Q(E) | 0.097% |
| Half-life | 0.068% |
| Weighing | 0.108% |
| Dilution factor | 0.019% |
| Sources & methods | 0.188% |
| Combined value | 0.330% |

**Isotopic ratio**

**Purpose:** Determination of the isotopic composition of ^168^Er enriched materials using Sector Field Inductively Coupled Plasma Mass Spectrometer (SF-ICP-MS).

**Method:** Target materials (^168^Er_2_O_3_: 98.0%, ISOFLEX, USA and 98.2% Trace Sciences Int., Canada) were analyzed using an Element II® SF-ICP-MS, by Thermo Fisher Scientific.

**Result:** The results of both enriched materials were in good agreement with their certificate of analysis (CoA) (Table S2).

**Table S2.** Comparison of the measured and CoA mass distribution values for ISOFLEX (98.0% ISOFLEX, USA) and TRACE ^168^Er enriched Er_2_O_3_ (98.2% Trace Sciences Int. Canada).

|  | **ISOFLEX** | |  | **TRACE** | |
| --- | --- | --- | --- | --- | --- |
| **Mass** | **Measured**  **(%)** | **CoA**  **(%)** | **Mass** | **Measured**  **(%)** | **CoA**  **(%)** |
| 162 | 0.005 | 0.02 | 162 | 0.003 | <0.06 |
| 164 | 0.02 | 0.03 | 164 | 0.02 | <0.06 |
| 166 | 0.37 | 0.35 | 166 | 0.38 | 0.35 |
| 167 | 0.90 | 0.90 | 167 | 0.93 | 0.76 |
| 168 | 98.05 | 98.00 | 168 | 97.99 | 98.20 |
| 170 | 0.65 | 0.70 | 170 | 0.67 | 0.69 |
|  |  |  |  |  |  |

**References**

1. Broda R. A review of the triple-to-double coincidence ratio (TDCR) method for standardizing radionuclides. *Appl Radiat Isot* (2003) **58**:585–594. doi:10.1016/S0969-8043(03)00056-3

2. Broda R, Cassette P, Kossert K. Radionuclide metrology using liquid scintillation counting. *Metrologia* (2007) **44**: doi:10.1088/0026-1394/44/4/S06

3. Nedjadi Y, Bailat C, Caffari Y, Cassette P, Bochud F. Set-up of a new TDCR counter at IRA-METAS. *Appl Radiat Isot* (2015) **97**:113–117. doi:10.1016/j.apradiso.2014.12.023

4. Nedjadi Y, Laedermann JP, Bochud F, Bailat C. On the reverse micelle effect in liquid scintillation counting. *Appl Radiat Isot* (2017) **125**:94–107. doi:10.1016/j.apradiso.2017.04.020

5. Nucléide-LARA, 2018. Available at: http://www.nucleide.org/Laraweb/index.php

6. https://www.nndc.bnl.gov/nudat2/(accessed on 17.12.2020).

7. Berger MJ. ESTAR, PSTAR AND ASTAR a PC package for calculating stopping powers and ranges of electrons, protons and helium ions. *IAEA-NDS-144* (1993)

8. Gümüs H, Kabadayi Ö. Practical calculations of stopping powers for intermediate energy electrons in some elemental solids. *Vacuum* (2010) **85**:245–252. doi:10.1016/j.vacuum.2010.06.004

9. Tan Z, Xia Y. Stopping power and mean free path for low-energy electrons in ten scintillators over energy range of 20-20,000eV. *Appl Radiat Isot* (2012) **70**:296–300. doi:10.1016/j.apradiso.2011.08.012

10. Grau Malonda A, Grau Carles A. The ionization quench factor in liquid-scintillation counting standardizations. *Appl Radiat Isot* (1999) **51**:183–188. doi:10.1016/S0969-8043(98)00179-1
